# Aromatase in human liver and its diseases

**DOI:** 10.1002/cam4.85

**Published:** 2013-04-30

**Authors:** Shuko Hata, Yasuhiro Miki, Ryoko Saito, Kazuyuki Ishida, Mika Watanabe, Hironobu Sasano

**Affiliations:** 1 Department of Pathology, Tohoku University Graduate School of Medicine2-1 Seiryo-machi, Aoba-ku, Sendai, Miyagi, 980-8575, Japan; 2 Department of Oral Pathology, Tohoku University Graduate School of Dentistry4-1 Seiryo-machi, Aoba-ku, Sendai, Miyagi, 980-8575, Japan; 3 Department of Pathology, Tohoku University Hospital1-1 Seiryou-machi, Aoba-ku, Sendai, Miyagi, 980-8576, Japan

**Keywords:** Liver cancer, metastasis, pathology

## Abstract

Estrogens play important roles in the cell proliferation and invasion of estrogen-dependent human neoplasms. Aromatase overexpression has been also reported in hepatitis and hepatocellular carcinoma (HCC) compared with normal liver but its details in these hepatic disorders have remained unclear. Therefore, in this study, we first immunolocalized aromatase using immunohistochemistry in patients with liver cirrhosis, steatosis, hepatitis, HCC, and metastasis liver carcinoma (MLC) in order to study the detailed status of intrahepatic aromatase. Aromatase immunoreactivity was predominantly detected in nonneoplastic hepatocytes around tumor cells. We then evaluated the effects of an interaction between hepatocytes and carcinoma cells upon aromatase mRNA expression, using HepG2 as a substitute model of hepatocytes by coculture systems. Aromatase mRNA levels in HepG2 were significantly increased by coculture with all carcinoma cell lines examined. We also evaluated alternative splicing of aromatase exon 1 but the same splicing variant was used in HepG2 cells regardless of carcinoma cell lines employed in the coculture system. These findings obtained in HepG2 indicated that carcinoma cells, whether metastatic or primary, induced aromatase expression in adjacent normal hepatocytes possibly through the soluble aromatase inducible factors in human hepatic microenvironments.

## Introduction

Estrogens play important roles in the growth and invasion of various estrogen-dependent neoplasms. Human liver is known as one of the target organs for estrogens, which have been proposed to function as mitogenic factor in both normal and diseased liver [1]. Differential expression of wild type and variant forms of estrogen receptor (ER) has been also reported in normal liver and hepatocellular carcinoma (HCC), chronic liver disease, indicating a possible link between sex hormones and pathogenesis of human liver diseases [2–6]. Circulating estrogens are predominantly hydroxylated by various metabolizing enzymes, such as CYP3A, 1A, and 1B in liver [7–9]. The expression of estrogen-metabolizing enzymes above was also reported to be decreased by hepatocyte damages associated with cirrhosis [2, 4, 6, 10]. This disruption of estrogen metabolism is in general considered the main pathogenesis of the development of gynecomastia in male patients with liver cirrhosis [2, 4, 6, 10]. Aromatase is a microsomal member of the cytochrome P450 superfamily, namely aromatase cytochrome P450 [11] and plays the pivotal roles in estrogen biosynthesis from circulating androgens [12]. In human liver diseases, the aromatase overexpression was first reported in fibrolamellar HCC cells of a 17-year-old male patient associated with marked gynecomastia [13]. Aromatase expression was also reported in hepatitis and HCC [10, 14]. In normal liver, aromatase was reported to be detected only in fetal hepatocytes but not in neonatal to adult liver [1, 15–19]. These results all indicated that aromatase inducible factors such as cytokines or prostaglandins may be produced in pathological liver microenvironment but its details have not been clarified yet at this juncture.

The status of intratumoral aromatase has been examined in details in breast cancer and other malignancies [1, 12, 15, 20, 21]. In human breast cancer tissue, aromatase in both stromal and parenchymal cells was reported to be regulated by various factors including “cell to cell interactions” and “cytokines signals” [22–24]. Therefore, the alterations of the intrahepatic microenvironment such as inflammation, fibrosis, and cancer metastasis are reasonably postulated to induce aromatase expression in nonpathological hepatocytes adjacent to these diseased cells. Therefore, in this study, we first immunolocalized aromatase in the patients with cirrhosis, steatosis, hepatitis, HCC, and metastasis liver carcinoma (MLC). In addition, Harada et al. [1] reported that hepatocytes around the tumor demonstrated relatively high expression of aromatase in metastatic carcinoma in the liver. Therefore, we also examined the possible effects of carcinoma cells upon aromatase mRNA expression in HepG2 using a coculture systems developed in our laboratory [24, 25]. In this study, we employed two different types of coculture system, namely “separation” and “contact” methods. We subsequently examined the expression patterns of liver specific aromatase promoter exon 1s (1b, I.5, and I.6), which plays very important roles in regulation of aromatase mRNA expression [16, 17], in HepG2 with or without the coculture with carcinoma cell lines by exon 1-specific RT-PCR analyses in order to further evaluate the regulation of intrahepatic aromatase. In addition, we examined expression levels of other aromatase promoter exon 1s (1a, 1c, and 1d) which could explain the overall increase in aromatase expression [17].

## Materials and Methods

### Patients and tissue preparation

The total of 65 cases with both nonneoplastic and neoplastic hepatic disorders (five cirrhosis, five steatosis, 26 hepatitis, six HCC, 23 MLC) were selected from surgical pathology files between 2003 and 2010 from Department of Pathology, Tohoku University Hospital (Sendai, Japan) after careful histopathological review of the cases. In 23 MLC cases, the primary lesions were as follows: adenocarcinoma of the lung (*n* = 2), stomach (*n* = 2), colon (*n* = 8), rectum (*n* = 3), gallbladder (*n* = 1), ovary (*n* = 2), and uterus (*n* = 1); squamous cell carcinoma of lung (*n* = 3); tubular adenocarcinoma of colon (*n* = 1). Among these 23 cases, 13 cases of primary carcinoma tissues (lung [*n* = 1], stomach [*n* = 2], colon [*n* = 6], rectum [*n* = 3], gallbladder [*n* = 1]) were available for examination. Relevant clinical data were also retrieved from the review of patients' files. The total of 12 cases of normal liver tissues were obtained from autopsy at the Department of Pathology, Tohoku University Hospital, Sendai, Japan. The ethics committees at Tohoku University School of Medicine approved these particular research protocols (2007-339, 2008-123).

### Immunohistochemistry

The antibodies used in this study were as follows: ERα (6F11; Novocastra Lab., Newcastle, U.K.), ERβ (14C8; GeneTex Inc., San Antonio, TX), and aromatase. The characteristic of primary monoclonal antibody for aromatase was described previously [24, 26, 27]. The primary antibody for aromatase #677 was kindly provided by Dr. Dean B. Evans (Novartis Institutes for BioMedical Research Basel, Oncology Research, Basel, Switzerland). The antigen–antibody complex was visualized with 3.3′-diaminobenzidine solution and counterstained with hematoxylin. Evaluation of immunohistochemistry was performed in 10% formalin-fixed and paraffin-embedded tissue specimens. Aromatase immunoreactivity was evaluated according to the immunointensity proportion scoring systems used for breast carcinoma tissues with some modifications [24, 26, 27]. The relative intensity of aromatase immunopositive cells was classified as follows: 0, no immunoreactivity; 1, weak; and 2, intense immunoreactivity. Results of immunohistochemistry were independently evaluated by two of the authors (S. H. and Y. M.). When evaluating ERs, more than 1000 cells from HCC, MLC, and primary tumors from same case were counted independently by the same two authors above and the percentage of immunoreactivity as a labeling index (LI in%) was subsequently determined [24].

### Cell lines

The cell lines used in the study of coculture system were HCC (HepG2), breast adenocarcinoma (MCF-7), lung adenocarcinoma (A549), and colon adenocarcinoma (DLD-1, HCT-15, WiDr, COLO205, SW480). The cell lines were obtained from Cell Resource Center for Biomedical Research, Tohoku University, Sendai, Japan and maintained in a RPMI-1640 (Sigma-Aldrich Co., St. Louis, Mo) with 10% fetal bovine serum.

### Coculture assay

For “separation” coculture, we employed 6 well ThinCert (Greiner Bio-One, Germany) culture insert with 4 μm pores membrane. HepG2 cells were cultured in six well plates in the absence or presence of carcinoma cell lines cultivated in coculture inserted. After 72 h of coculture, HepG2 cells were carefully separated and the levels of aromatase mRNA were examined by quantitative reverse transcription polymerase chain reaction (RT-PCR).

In “contact” coculture, we employed cloning ring and membrane slide used for laser capture microdissection (LCM). Two rings of different diameter (ø150 mm and ø100 mm, IWAKI, Japan) were bonded to the LCM membrane with the state of the nest (Fig. 5A). Carcinoma cell lines were cultured inside of this inner ring above and the HepG2 cells were cultured between inner ring and outer ring, respectively. The inner ring was removed in 24 h after the cell culture. Following 7 days of coculture, HepG2 cells, which had contacted with carcinoma cells, were carefully separated by LCM. LCM was performed using MMI Cellcut (Molecular Machines & Industries, Flughofstrase, Glattbrugg, Switzerland). Total RNA was extracted from LCM subjects using the RNeasy Micro Kit (Qiagen Inc., Mississauga, Ontario, Canada) for real-time PCR. Direct physical cell to cell contact occurred in “contact” coculture but not in “separation” one.

### Total RNA extraction from HepG2 cell coculture and cDNA synthesis

Total RNA was carefully extracted from control-HepG2 and coculture-HepG2 using the TRIzol method (Invitrogen Corporation, San Diego, CA) [28]. A reverse transcription kit (QuantiTect Reverse Transcription; Qiagen) was employed in the synthesis of cDNA.

### Quantitative RT-PCR

Quantitative RT-PCR was carried out using the LightCycler System (Roche Diagnostics GmbH, Mannheim, Germany). The primer positions used in this study were summarized as follows: *RPL13A* (NM_012423), forward 487–reverse 612 [29]; *aromatase* (X13589), forward 691–reverse 806 [24]; *ERα* (NM_000125), forward 1811–reverse 2100 [30]; *ERβ* (AB006590), forward 1460–reverse 1627 [30]. *Aromatase* mRNA levels in each case were represented as a ratio of *RPL13A* and evaluated as a percentage ratio [29, 31]. cDNA of known concentrations for target genes and the housekeeping gene, ribosomal protein L13a (*RPL13A*) were used to generate standard curves for real-time quantitative PCR to determine the quantity of target cDNA transcripts. The mRNA level in each case was represented as a ratio of *RPL13A* (%) [29].

### Analysis of aromatase exon 1 expression

Following 72 h of cultivation using this “separation” coculture system above, HepG2 cells were carefully separated and the expressions of aromatase exon 1 were examined by RT-PCR. PCR was carried out using the MJ Research PTC-200 Peltier Thermal Cycler (Bio-Rad, Hercules, CA). The reaction used Phusion High-Fidelity DNA Polymerase (FINNZYMES, Espoo, Finland). PCR conditions were 98°C for 30 seconds, 30 cycles of 98°C for 10 seconds, 55°C for 30 seconds, and 72°C for 30 seconds, followed by 72°C for 5 minutes. PCR products were then separated by electrophoresis on 2% agarose gels. Sequences of oligonucleotides used for amplification and construction is indicated in Table 1 [17, 32].

**Table 1:** Sequences of oligonucleotides primer used by PCR amplification

Primer	Oligonucleotide sequence	Reference
Exon 1a	5′-GTG GAG GCA AAA CAG GAA GGT GAA GAA GAA C-3′	[17]
Exon 1b	5′-GTA GAA CGT GAC CAC TGG-3′	[17]
Exon 1c	5′-GAT AAG GTT CTA TCA GAC C-3′	[17]
Exon 1d	5′-GCA ACA GGA GCT ATA GAT GAA CCT T-3′	[17]
Exon I.5	5′-TTC CTG TCT CCA GAT TGG CTG GGA-3′	[17]
Exon I.6	5′-GAG CAG CTA ACG TCT GTG CAA-3′	[32]
Exon III	5′-CAC CCG GTT GTA GTA GTT GCA GGC ACT GCC-3′	[17]

### Statistical analysis

Statistical analysis was performed using the StatView 5.0 J software (SAS Institute Inc., Cary, NC). Immunohistochemical scores and coculture assay data were analyzed by analysis of variance followed by post hoc Bonferroni/Dunnet multiple comparison tests. A *P*-value <0.05 was considered to indicate statistical significance.

## Results

### Immunolocalization of aromatase and ERs

**Figure 1:**
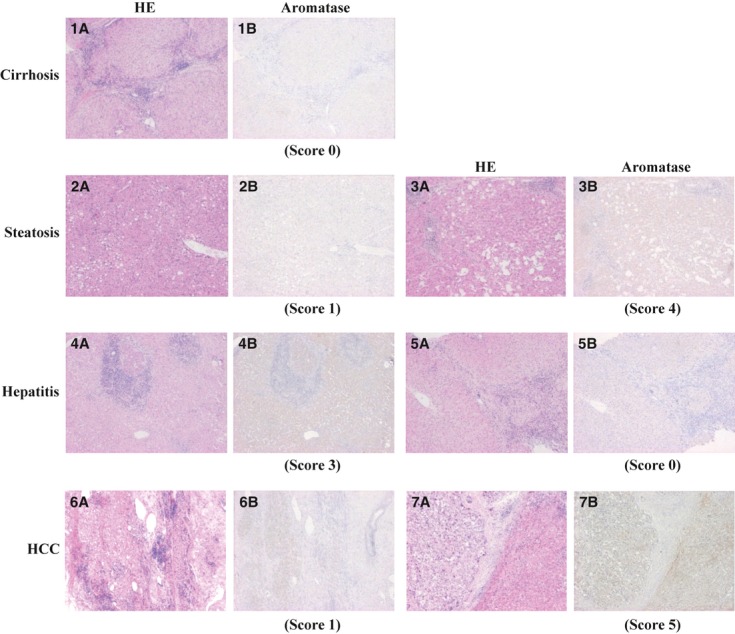
Immunolocalization of aromatase in representative histological sections of the liver diseases. (A) Hematoxylin–eosin-stained tissue sections. (B) Immunohistochemistry for aromatase in liver diseases. 1A and 1B, cirrhosis; 2A–3B, steatosis; 4A–5B, hepatitis; 6A–7B, HCC.

**Figure 2:**
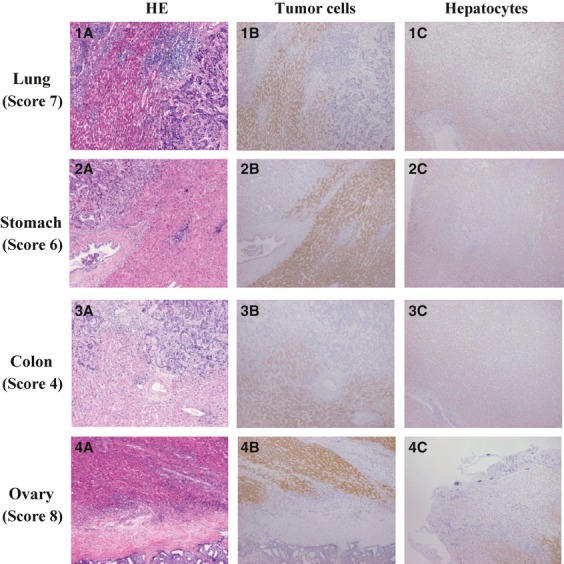
Immunolocalization of aromatase in representative histological sections of the metastatic liver carcinoma. (A) Hematoxylin–eosin-stained tissue sections. (B) Immunohistochemistry for aromatase in metastatic liver carcinoma (tumor cells). (C) Immunohistochemistry for aromatase in metastatic liver carcinoma (hepatocytes). 1A–1C, lung carcinoma; 2A–2C, stomach carcinoma; 3A–3C, colon carcinoma; 4A–4C, ovary carcinoma.

**Figure 3:**
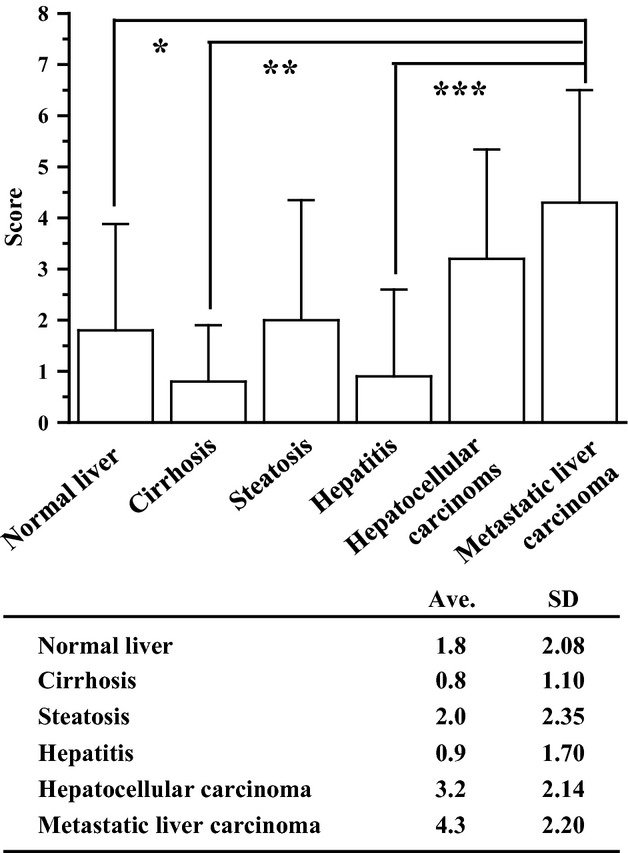
Results of immunohistochemistry scoring of aromatase expression in patients with normal liver and liver diseases. *P = 0.0009; **P = 0.0007; ***P < 0.0001.

Representative findings of aromatase immunohistochemistry in human liver diseases were illustrated in Figures 1 and 2. The scores of aromatase immunohistochemistry (aromatase scores) in each liver disease were also summarized in Figure 3. The aromatase immunoreactivity was detected in the cytoplasm of normal hepatocytes and hepatic tumor cells but not in Kupffer and/or stromal cells including fibroblasts. The aromatase score (mean ± SD) was relatively low in the hepatocytes of cirrhosis (0.8 ± 1.10), steatosis (2.0 ± 2.35), and hepatitis (0.9 ± 1.70) as well as of normal or nonpathological liver (1.8 ± 2.08). Relatively high aromatase score was detected in HCC cases (3.2 ± 2.14), but the differences did not reach statistical significance. In MLC cases examined, the aromatase score (4.3 ± 2.20) was significantly higher than that in normal liver (*P* = 0.0009), cirrhosis (*P* = 0.0007), and hepatitis (*P* < 0.0001).

In MLC cases, aromatase immunoreactivity was predominantly detected in the cytoplasmic compartment of hepatocytes adjacent to the sites of carcinoma infiltration. However, aromatase immunoreactivity was hardly detected in either tumor cells or in the hepatocytes away from metastatic carcinoma infiltration. Aromatase immunoreactivity was not detected in two case of MLC (lung squamous cell carcinoma and rectum adenocarcinoma) examined. There were no significant differences of aromatase scores among the different primaries in these cases of MLC examined in this study.

**Figure 4:**
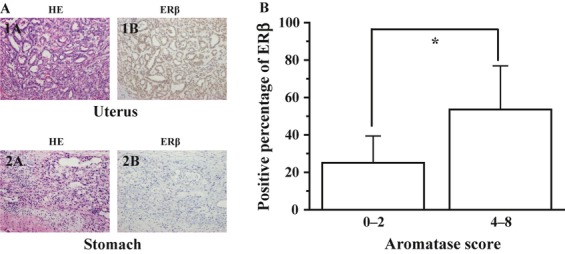
Immunolocalization of ERβ in metastatic liver carcinoma. (A) Immunolocalization of ERβ in representative histological sections of the metastatic liver carcinoma. (a) Hematoxylin–eosin-stained tissue sections. (b) Immunohistochemistry for ERβ in metastatic liver carcinoma. 1A and 2A, uterus carcinoma, High-expression case of ERβ; 2A and 2B, stomach carcinoma, Low-expression case of ERβ. (B) Results of immunohistochemistry scoring of ERβ expression in patients with metastatic liver carcinoma in aromatase scoring. *P = 0.011.

ERβ immunoreactivity was predominantly detected in the nuclei of hepatocytes and tumor cells as illustrated in Figure 4A. In MLC cases examined, ERβ LI was significantly higher in high aromatase score cases (score 4–8) than low aromatase score cases (score 0–2) (Fig. 4C) (*P* < 0.011). ERβ LI in primary sites of the same patients was significantly higher than that in MLCs (*P* < 0.0025). ERα was not detectable in both normal hepatocytes and tumor in MLC and its primary tumors cells examined.

### Effects of coculture with carcinoma cells on aromatase mRNA expression in HepG2

Results of “separation” coculture of carcinoma cell lines on the HepG2 aromatase mRNA were summarized in Table 2. Aromatase mRNA level in HepG2 was significantly increased by cocultivation with all carcinoma cell lines examined in this study. Aromatase mRNA level in HepG2 was significantly increased particularly by cocultivation with WiDr cell (expression of ratio, coculture/control; 511%). The levels of ERα and ERβ mRNA evaluated by quantitative RT-PCR were also summarized in Table 2. ERα was detected in MCF-7 and WiDr cells and ERβ in all carcinoma cell lines was examined. Relatively high level of ERβ expression was detected in WiDr cells.

**Table 2:** Summary of cell line and qRT-PCR of aromatase expression in coculture assay

		ER^1^ mRNA (% of RPL13A)		Coculture/control		
			
Coculture cells	Primary tissue	ERα	ERβ	Aromatase expression (%)	SD	*P*-value
MCF-7	Breast adenocarcinoma	0.3271702	0.0000471	161	0.002	0.0044
A549	Lung adenocarcinoma	–	0.0000416	275	0.003	0.0005
DLD-1	Colon adenocarcinoma	–	0.0000460	378	0.549	0.0006
WiDr	Colon adenocarcinoma	0.0015806	0.0000732	511	0.286	<0.0001
HCT-15	Colon adenocarcinoma	–	0.0000237	269	0.003	0.0011
COLO205	Colon adenocarcinoma	–	0.0000459	147	0.002	0.0013
SW480	Colon adenocarcinoma	–	0.0000143	160	0.006	0.0400

–, under detectable level.

1 The ERα and β of mRNA level in each carcinoma cell monocultured as a ratio of RPL13A.

**Figure 5:**
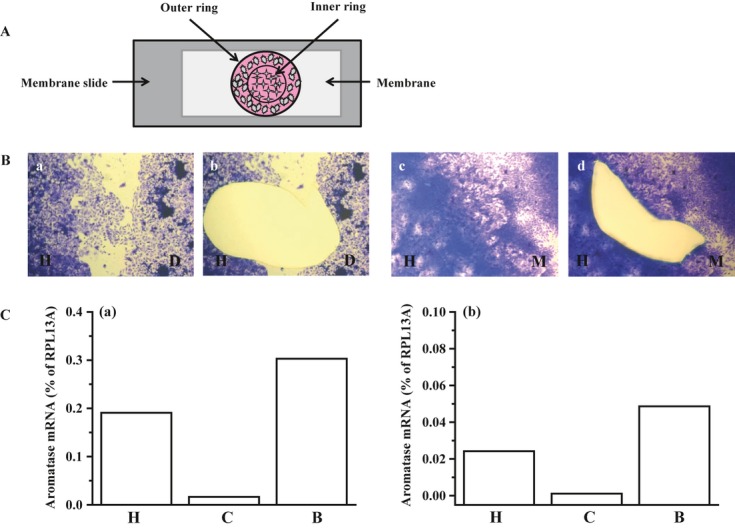
Expression of aromatase in coculture/LCM HepG2. (A) Coculture/LCM model system. (B) Results of LCM/qPCR for aromatase in cocultured HepG2 on the membrane. Coculture was examined by toluidine blue staining. a, before cutting boundary region of HepG2 and DLD-1; b, after cutting boundary region of HepG2 and DLD-1; c, before cutting boundary region of HepG2 and MCF-7; d, after cutting boundary region of HepG2 and MCF-7. H, HepG2 region; D, DLD-1 region; M, MCF-7 region. (C) Results of LCM/qPCR for aromatase expression in cocultured HepG2. (a) cocultured with DLD-1; (b) cocultured with MCF-7. H, only HepG2 region; C, only cancer cell line region; B, boundary region of HepG2.

Results of “contact” coculture of carcinoma cell lines on the HepG2 aromatase mRNA were summarized in Figure 5. The HepG2 cells in the area which physically contacted with MCF-7 and DLD-1 were separated by LCM (Fig. 5B). The areas of HepG2 cells not physically contact with carcinoma cells and carcinoma cells themselves were also isolated with LCM (Fig. 5B). The expression level of aromatase mRNA was relatively high in the area of HepG2 in direct physical contact with both MCF-7 and DLD-1 compared to no-contacted HepG2 (Fig. 5C). The aromatase mRNA in carcinoma cell lines, MCF-7 and DLD-1, was markedly low (Fig. 5C).

### Analysis of exon 1 in cocultured HepG2

**Figure 6:**
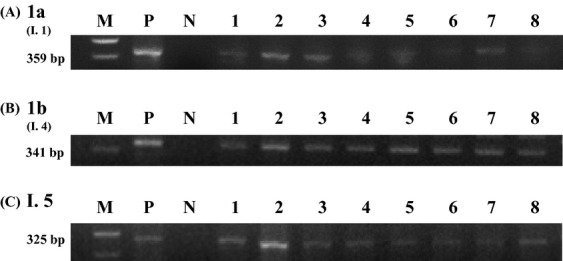
Amplified products obtained by PCR of RNA extracted from coculture HepG2. PCR was performed using oligonucleotides hybridizing to regions of the exons indicated in each row (A–C). The estimated sizes of the amplified products are indicated on the left. Coculture were obtained from: lane P, positive control; lane N, negative control; lane 1, HepG2; lane 2, cocultured HepG2 by MCF-7; lane 3, cocultured HepG2 by A549; lane 4, cocultured HepG2 by DLD-1; lane 5, cocultured HepG2 by WiDr; lane 6, cocultured HepG2 by HCT-15; lane 7, cocultured HepG2 by COLO205; lane 8, cocultured HepG2 by SW480.

Results were summarized in Figure 6. We examined expression levels of liver specific exon 1s (1b, I.5, and I.6) and other exon 1s (1a, 1c, and 1d) which may be related to the overall increase in aromatase expression. The amplifications of exon 1a, 1b, and I.5 were all detected in monocultured HepG2 as well as fetal liver tissues examined as a positive control. There were no significant differences of the status of aromatase exon 1a, 1b, and I.5 of HepG2 studied between mono and coculture conditions. Relatively high expression of aromatase exon 1a, 1b, and I.5 was detected in HepG2 cells cocultured with MCF-7. Aromatase promotor exon 1c, 1d, and I.6 in mono/coculture of HepG2 as well as fetal liver were below the detection levels in this study (data not present).

## Discussion

In adult human liver, aromatase has been reported to be nearly undetectable in normal hepatocytes [10, 14], but increased aromatase expression and activity, along with locally elevated estrogen levels, has been reported in nonmalignant tissues from liver with alcoholic cirrhosis [14]. Several CYP proteins were also reported to be downregulated by inflammatory cytokines such as interleukin-1 (IL-1), IL-6, tumor necrosis factor α (TNFα), interferon, and transforming growth factor β (TGFβ) in human hepatocytes [33, 34]. In addition, prostaglandin E2 and cytokines, such as IL-6 or TNFα, were also reported to regulate CYP19A1 expression and aromatase activity [6, 22, 23]. Therefore, the intrahepatic level of aromatase may be reasonably postulated to be influenced by that of the factors above, which is different among several types of inflammatory or neoplastic disease such as hepatitis, cirrhosis, steatosis, HCC, and MLC [35]. However, in our present study, no significant aromatase immunoreactivity was detected in the adjacent normal hepatocytes with possible exceptions of HCC and MLC. These results suggest that cytokines or other factors derived from the process of hepatic neoplasms, not benign inflammatory conditions such as cirrhosis, including metastatic liver tumors can induce intrahepatic aromatase in a more efficient manner. Harada et al. [1] previously detected aromatase in seven cases of liver metastasis from gastrointestinal carcinoma. We also recently reported that in the other CYP family, CYP2E1 immunoreactivity was markedly detected in hepatocytes around the tumor of MLC tissues compared to hepatocytes located in distant areas from the tumor in human liver [36]. Results of these studies above as well as those from our present investigation all indicated that the tumor cells in both HCC and MLC may secrete potent inducers of CYP19A1 into adjacent microenvironment of the human liver, which resulted in an induction of intrahepatic aromatase in hepatocytes.

In this study, we first evaluated possible effects of interactions of carcinoma cells upon aromatase mRNA expression in HepG2 using a coculture systems of “separation.” The HCC cell line, HepG2, has been widely used as a substitute for “liver cell” in many previous in vitro studies and because it is relatively easy to evaluate compared to primary human hepatocytes. However, it has also been considered difficult to evaluate the normal liver metabolism in the study employing HepG2 cells as an in vitro model, because the expression of drug-metabolizing enzymes is relatively low compared to primary normal hepatocytes [37, 38]. In addition, in our previous study [36], we examined the gene expression profiles of phase I metabolic enzymes including CYP19A1 using quantitative RT-PCR array (SuperArray System, Qiagen) and demonstrated that the expression profiles of HepG2 were indeed markedly different from those of normal human liver tissues. However, it is also true that the expression level of CYP19A1 in HepG2 was relatively low as well as in adult normal liver tissues compared with those levels in fetal livers. In addition, we also demonstrated that expression patterns of aromatase exon 1 in HepG2 cells were the same as in normal liver in our present study. Therefore, we decided to use HepG2 cells as the in vitro model substitute for normal hepatocytes in our present study, which subsequently demonstrated that aromatase expression was induced by cell to cell interaction. This is the first study to demonstrate effects of intrahepatic aromatase mRNA expression by interactions with the different carcinoma cell lines derived from primary tumors of the liver metastasis. The level of aromatase mRNA expression in HepG2 cells was significantly increased by “separation” coculture with various carcinoma cell lines examined. In primary breast cancer tissue environment, aromatase was known to be predominantly detected in the fibroblastic stromal cells and increased by soluble factors derived from carcinoma cells [24]. Therefore, these carcinoma cells which metastasized to the liver may induce aromatase and provide the estrogens through cell to cell interactions in liver microenvironment. The “direct” cell to cell contact is important to examine the immune and neuronal functions in vitro [39, 40]. However, it is true that albumin and urea were not significantly different between “direct” and “separation” coculture of hepatocyte derived from adult and fetal mouse [41]. Therefore, in this study, we also examined the effects of direct contact with carcinoma cells upon aromatase expression in HepG2 cells using “contact” coculture system. This is the first study to demonstrate aromatase mRNA expression in the “contact” of coculture/LCM of carcinoma cell lines following isolation using laser capture microscopy and subsequent qRT-PCR analysis. In this analysis, there were no significantly differences between the HepG2 located in contact areas and in distant areas in which no physical contact between carcinoma cell lines and HepG2 cells were detected. These results from “separation” and “contact” coculture analysis did demonstrate that an induction of aromatase in HepG2 cells by carcinoma cell lines could predominantly depend upon soluble factors secreted from carcinoma cells but not direct physical “cell to cell contact” [22–24] as in the results of coculture study of breast carcinoma cells [24]. In our present study, HepG2, MCF-7, and DLD-1 all secreted aromatase inducible cytokines such as IL-1, IL-6, and oncostatin M (data not present). Therefore, aromatase expression in HepG2 under coculture conditions may be induced by ILs derived from both carcinoma cells (paracrine) and HepG2 itself (autocrine), respectively. Further studies using HCC or normal liver-derived cell lines are definitively required to clarify the aromatase expression induced by cell to cell interactions in normal human hepatocytes.

It is well known that among metastatic liver cancers, primary tumors were predominantly from the lung, colon, pancreas, breast, and stomach in autopsy series [40]. Ovarian and endometrial carcinomas, estrogen-dependent malignancies, are less frequent than these malignancies above among MLC [42]. Therefore, in this study, we examined the expression and localization of aromatase protein in MLC from carcinoma in the lung, stomach, colorectum, gallbladder, ovary, and endometrium. Many adenocarcinoma cases of the breast, endometrium, and ovary express ERα and are estrogen dependent for their growth. However, results of several studies also suggested that lung carcinoma might be one of the estrogen-dependent cancer [25, 43, 44]. There have been controversies regarding the roles of estrogen on carcinomas of the gastrointestinal tracts [45, 46]. However, it is true that ERα and/or ERβ were reported to be expressed in gastrointestinal carcinomas including colorectum and gastric carcinomas [46–48]. Therefore, the carcinomas metastasized to liver influence the intrahepatic microenvironment and may augment the estrogenic actions on carcinoma cells themselves in an intracrine fashion. In breast cancer, the disconcordance or discrepancies have been reported in the status of ERs between primary and metastatic tumors [49]. We also detected the decreased ERβ LI in MLC compared with that in primary tumor but it awaits further investigations for clarification.

Markedly high level of aromatase expression was reported in human fetal liver, whereas hepatic aromatase expression normally becomes undetectable in postnatal life [16–19]. Exon 1b, I.5, and I.6 promoter of the aromatase gene containing sequence was reported to be present in RT-PCR amplified products in human fetal liver [16, 17, 50]. Agarwal et al. [13] demonstrated that aromatase exon 1b could not be detected in fibrolamellar HCCs. Aromatase promoter exon I.6 in HCC was reported to be detected by several investigators [35, 50]. These findings all indicated that the aromatase exon 1 utilization patterns may be changed by neoplastic transformation of hepatocytes themselves. However, in our present study, the patterns of expression or utilization of aromatase exon 1b and I.5 HepG2 cells were the same as that of fetal liver regardless of monoculture or coculture with various carcinoma cells. Therefore, increased aromatase mRNA in HepG2 cocultured with carcinoma cells is considered not to be related to the transformation of regulatory mechanisms of aromatase mRNA expression in HepG2 cells.

## References

[1] Harada N, Ota H, Yoshimura N, Katsuyama T, Takagi Y (1998). Localized aberrant expression of cytochrome P450 aromatase in primary and metastatic malignant tumors of human liver. J. Clin. Endocrinol. Metab.

[2] Nagasue N, Ito A, Yukaya H, Ogawa Y (1986). Estrogen receptors in hepatocellular carcinoma. Cancer.

[3] Villa E, Dugani A, Moles A, Camellini L, Grottola A, Buttafoco P (1998). Variant liver estrogen receptor transcripts already occur at an early stage of chronic liver disease. Hepatology.

[4] Iavarone M, Lampertico P, Seletti C, Francesca Donato M, Ronchi G (2003). The clinical and pathogenetic significance of estrogen receptor-beta expression in chronic liver diseases and liver carcinoma. Cancer.

[5] Kalra M, Mayes J, Assefa S, Kaul AK, Kaul R (2008). Role of sex steroid receptors in pathobiology of hepatocellular carcinoma. World J. Gastroenterol.

[6] Villa E (2008). Role of estrogen in liver cancer. Womens Health (Lond. Engl.).

[7] Stresser DM, Kupfer D (1998). Prosubstrates of CYP3A4, the major human hepatic cytochrome P450: transformation into substrates by other P450 isoforms. Biochem. Pharmacol.

[8] Yamazaki H, Shaw PM, Guengerich FP, Shimada T (1998). Roles of cytochromes P450 1A2 and 3A4 in the oxidation of estradiol and estrone in human liver microsomes. Chem. Res. Toxicol.

[9] Cribb AE, Knight MJ, Dryer D, Guernsey J, Hender K, Tesch M (2006). Role of polymorphic human cytochrome P450 enzymes in estrone oxidation. Cancer Epidemiol. Biomarkers Prev.

[10] Castagnetta LA, Agostara B, Montalto G, Polito L, Campisi I, Saetta A (2003). Local estrogen formation by nontumoral, cirrhotic, and malignant human liver tissues and cells. Cancer Res.

[11] Simpson ER, Davis SR (2001). Minireview: aromatase and the regulation of estrogen biosynthesis – some new perspectives. Endocrinology.

[12] Harada N (1999). Aromatase and intracrinology of estrogen in hormone-dependent tumors. Oncology.

[13] Agarwal VR, Takayama K, Sasano JJ, Van Wyk H, Simpson ER, Bulun SE (1998). Molecular basis of severe gynecomastia associated with aromatase expression in a fibrolamellar hepatocellular carcinoma. J. Clin. Endocrinol. Metab.

[14] Granata OM, Cocciadifero L, Campisi I, Miceli V, Montalto G, Polito LM (2009). Androgen metabolism and biotransformation in nontumoral and malignant human liver tissues and cells. J. Steroid Biochem. Mol. Biol.

[15] Harada N, Utsumi T, Takagi Y (1993). Tissue-specific expression of the human aromatase cytochrome P-450 gene by alternative use of multiple exons 1 and promoters, and switching of tissue-specific exons 1 in carcinogenesis. Proc. Natl. Acad. Sci. USA.

[16] Zhao Y, Mendelson CR, Simpson ER (1995). Characterization of the sequences of the human CYP19 (aromatase) gene that mediate regulation by glucocorticoids in adipose stromal cells and fetal hepatocytes. Mol. Endocrinol.

[17] Toda K, Simpson ER, Mendelson CR, Shizuta Y, Kilgore MW (1994). Expression of the gene encoding aromatase cytochrome P450 (CYP19) in fetal tissues. Mol. Endocrinol.

[18] Yamamoto T, Sakai C, Yamaki J, Takamori K, Yoshiji S, Kitawaki J (1984). Estrogen biosynthesis in human liver – a comparison of aromatase activity for C-19 steroids in fetal liver, adult liver and hepatoma tissues of human subjects. Endocrinol. Jpn.

[19] Price T, Aitken J, Simpson ER (1992). Relative expression of aromatase cytochrome P450 in human fetal tissues as determined by competitive polymerase chain reaction amplification. J. Clin. Endocrinol. Metab.

[20] Sasano H, Harada N (1998). Intratumoral aromatase in human breast, endometrial, and ovarian malignancies. Endocr. Rev.

[21] Bulun SE, Lin Z, Imir G, Amin S, Demura M, Yilmaz B (2005). Regulation of aromatase expression in estrogen-responsive breast and uterine disease: from bench to treatment. Pharmacol. Rev.

[22] Zhao Y, Agarwal VR, Mendelson CR, Simpson ER (1996). Estrogen biosynthesis proximal to a breast tumor is stimulated by PGE2 via cyclic AMP, leading to activation of promoter II of the CYP19 (aromatase) gene. Endocrinology.

[23] Zhou J, Suzuki T, Kovacic A, Saito R, Miki Y, Ishida T (2005). Interactions between prostaglandin E(2), liver receptor homologue-1, and aromatase in breast cancer. Cancer Res.

[24] Miki Y, Suzuki T, Tazawa C, Yamaguchi Y, Kitada K, Honma S (2007). Aromatase localization in human breast cancer tissues: possible interactions between intratumoral stromal and parenchymal cells. Cancer Res.

[25] Miki Y, Suzuki T, Abe K, Suzuki S, Niikawa H, Iida S (2010). Intratumoral localization of aromatase and interaction between stromal and parenchymal cells in the non-small cell lung carcinoma microenvironment. Cancer Res.

[26] Sasano H, Edwards DP, Anderson TJ, Silverberg SG, Evans DB, Santen RJ (2003). Validation of new aromatase monoclonal antibodies for immunohistochemistry: progress report. J. Steroid Biochem. Mol. Biol.

[27] Lykkesfeldt AE, Henriksen KL, Rasmussen BB, Sasano H, Evans DB, Moller S (2009). In situ aromatase expression in primary tumor is associated with estrogen receptor expression but is not predictive of response to endocrine therapy in advanced breast cancer. BMC Cancer.

[28] Chomczynski P, Sacchi N (1987). Single-step method of RNA isolation by acid guanidinium thiocyanate-phenol-chloroform extraction. Anal. Biochem.

[29] Suzuki T, Miki Y, Moriya T, Shimada N, Ishida T, Hirakawa H (2004). Estrogen-related receptor alpha in human breast carcinoma as a potent prognostic factor. Cancer Res.

[30] Niikawa H, Suzuki T, Miki Y, Suzuki S, Nagasaki S, Akahira J (2008). Intratumoral estrogens and estrogen receptors in human non-small cell lung carcinoma. Clin. Cancer Res.

[31] Suzuki T, Nakata T, Miki Y, Kaneko C, Moriya T, Ishida T (2003). Estrogen sulfotransferase and steroid sulfatase in human breast carcinoma. Cancer Res.

[32] Shozu M, Zhao Y, Simpson ER (1997). Estrogen biosynthesis in THP1 cells is regulated by promoter switching of the aromatase (CYP19) gene. Endocrinology.

[33] Muntane-Relat J, Ourlin JC, Domergue J, Maurel P (1995). Differential effects of cytokines on the inducible expression of CYP1A1, CYP1A2, and CYP3A4 in human hepatocytes in primary culture. Hepatology.

[34] Aitken AE, Morgan ET (2007). Gene-specific effects of inflammatory cytokines on cytochrome P450 2C, 2B6 and 3A4 mRNA levels in human hepatocytes. Drug Metab Dispos.

[35] Koh WP, Yuan JM, Wang R, Govindarajan S, Oppenheimer R, Zhang ZQ (2011). Aromatase (CYP19) promoter gene polymorphism and risk of nonviral hepatitis-related hepatocellular carcinoma. Cancer.

[36] Hata S, Miki Y, Fujishima F, Sato R, Okaue A, Abe K (2010). Cytochrome 3A and 2E1 in human liver tissue: individual variations among normal Japanese subjects. Life Sci.

[37] Wilkening S, Stahl F, Bader A (2003). Comparison of primary human hepatocytes and hepatoma cell line Hepg2 with regard to their biotransformation properties. Drug Metab Dispos.

[38] Westerink WM, Schoonen WG (2007). Cytochrome P450 enzyme levels in HepG2 cells and cryopreserved primary human hepatocytes and their induction in HepG2 cells. Toxicol. In Vitro.

[39] Guarini A, Riera L, Cignetti A, Montacchini L, Massaia M, Foa R (1997). Transfer of the interleukin-2 gene into human cancer cells induces specific antitumor recognition and restores the expression of CD3/T-cell receptor associated signal transduction molecules. Blood.

[40] Tieu K, Ashe PC, Zuo DM, Yu PH (2001). Inhibition of 6-hydroxydopamine-induced p53 expression and survival of neuroblastoma cells following interaction with astrocytes. Neuroscience.

[41] Van Poll D, Sokmensuer C, Ahmad N, Tilles AW, Berthiaume F, Toner M (2006). Elevated hepatocyte-specific functions in fetal rat hepatocytes co-cultured with adult rat hepatocytes. Tissue Eng.

[42] Centeno BA (2006). Pathology of liver metastases. Cancer Control.

[43] Weinberg OK, Marquez Garban DC, Fishbein MC, Goodglick L, Garban HJ, Dubinett SM (2005). Aromatase inhibitors in human lung cancer therapy. Cancer Res.

[44] Miki Y, Abe K, Suzuki S, Suzuki T, Sasano H (2011). Suppression of estrogen actions in human lung cancer. Mol. Cell. Endocrinol.

[45] Deroo BJ, Korach KS (2006). Estrogen receptors and human disease. J. Clin. Invest.

[46] Hogan AM, Collins D, Baird AW, Winter DC (2009). Estrogen and gastrointestinal malignancy. Mol. Cell. Endocrinol.

[47] Taylor AH, Al Azzawi F (2000). Immunolocalisation of oestrogen receptor beta in human tissues. J. Mol. Endocrinol.

[48] Jiang H, Teng R, Wang Q, Zhang X, Wang H, Wang Z (2008). Transcriptional analysis of estrogen receptor alpha variant mRNAs in colorectal cancers and their matched normal colorectal tissues. J. Steroid Biochem. Mol. Biol.

[49] Aitken SJ, Thomas JS, Langdon SP, Harrison DJ, Faratian D (2010). Quantitative analysis of changes in ER, PR and HER2 expression in primary breast cancer and paired nodal metastases. Ann. Oncol.

[50] Shozu M, Zhao Y, Bulun SE, Simpson ER (1998). Multiple splicing events involved in regulation of human aromatase expression by a novel promoter, I.6. Endocrinology.

